# Adiponectin as a predictor of mortality and readmission in patients with community-acquired pneumonia: a prospective cohort study

**DOI:** 10.3389/fmed.2024.1329417

**Published:** 2024-04-02

**Authors:** Arnold Matovu Dungu, Camilla Koch Ryrsø, Maria Hein Hegelund, Adin Sejdic, Andreas Vestergaard Jensen, Peter Lommer Kristensen, Rikke Krogh-Madsen, Daniel Faurholt-Jepsen, Birgitte Lindegaard

**Affiliations:** ^1^Department of Pulmonary and Infectious Diseases, Copenhagen University Hospital - North Zealand, Hilleroed, Denmark; ^2^Department of Endocrinology and Nephrology, Copenhagen University Hospital - North Zealand, Hilleroed, Denmark; ^3^Department of Clinical Medicine, University of Copenhagen, Copenhagen, Denmark; ^4^Department of Infectious Diseases, Copenhagen University Hospital - Hvidovre, Copenhagen, Denmark; ^5^Department of Infectious Diseases, Copenhagen University Hospital – Rigshospitalet, Copenhagen, Denmark

**Keywords:** community-acquired pneumonia, adiponectin, body mass index, mortality, readmission

## Abstract

**Background:**

Adiponectin is secreted by adipocytes and is inversely associated with obesity. Given the association between low body mass index (BMI) and higher mortality risk after community-acquired pneumonia (CAP), we hypothesized that high adiponectin levels are associated with a higher risk of adverse clinical outcomes in patients with CAP.

**Methods:**

In a prospective cohort study of 502 patients hospitalized with CAP, adiponectin was measured in serum at admission. The associations between adiponectin and clinical outcomes were estimated with logistic regression analyses adjusted for age, sex, and measures of obesity (BMI, waist circumference or body fat percentage).

**Results:**

Adiponectin was associated with higher 90-day mortality for each 1 μg/mL increase [OR 1.02, 95% CI (1.00, 1.04), *p* = 0.048] independent of age and sex. Likewise, adiponectin was associated with a higher risk of 90-day readmission for each 1 μg/mL increase [OR 1.02, 95% CI (1.01, 1.04), *p* = 0.007] independent of age and sex. The association between adiponectin and 90-day mortality disappeared, while the association with 90-day readmission remained after adjusting for adiposity.

**Conclusion:**

Adiponectin was positively associated with mortality and readmission. The association with mortality depended on low body fat, whereas the association with readmission risk was independent of obesity.

## 1 Introduction

Community-acquired pneumonia (CAP) is a significant cause of morbidity and mortality worldwide, with 90-day mortality rates up to 22% (1–4). Obese and underweight individuals have an increased risk of acquiring CAP (5, 6), whereas underweight patients with CAP have a higher mortality risk than normal-weight, overweight, and obese patients (7).

Individuals with obesity have increased fat and muscle mass, whereas underweight individuals have reduced fat and muscle mass (8). Adipose tissue secretes several adipokines, including adiponectin, with metabolic and immunological functions (9–11). Adiponectin is almost exclusively secreted by adipocytes (12). Mice studies have shown that adiponectin exerts several beneficial effects, including anti-inflammatory, anti-atherogenic, anti-apoptotic, and insulin-sensitizing effects (11). In humans, increasing age and female sex are associated with higher adiponectin levels, while obesity, type 2 diabetes mellitus and insulin resistance are associated with lower adiponectin levels (9). In addition, progressive weight loss increases adiponectin levels after bariatric surgery, caloric restriction (13, 14) or cachexia associated with chronic diseases (15–17).

Given the beneficial effects shown in mice studies, high adiponectin levels have been paradoxically associated with increased mortality from chronic diseases, including cardiovascular diseases, chronic obstructive pulmonary disease (COPD), cancer, and diabetes mellitus in epidemiological studies (18). However, the role of adiponectin in acute infections is incompletely understood with conflicting data as both low (19) and high (20, 21) adiponectin levels have been associated with higher sepsis-related mortality.

Given the inverse association between adiponectin and body mass index (BMI) (9) and the association between low BMI and increased mortality risk after CAP (7), we hypothesized that high levels of adiponectin are associated with an increased risk of adverse clinical outcomes in patients hospitalized with CAP. Therefore, this study aimed to assess the association between serum adiponectin levels and adverse clinical outcomes and predictors of adiponectin levels at admission in hospitalized patients with CAP.

## 2 Methods

### 2.1 Reporting

The STROBE (Strengthening the Reporting of Observational Studies in Epidemiology) guidelines for reporting observational cohort studies were followed when reporting this study (22).

### 2.2 Design and setting

This study used the Surviving Pneumonia research platform. Surviving pneumonia is an observational, prospective cohort study including hospitalized patients with CAP at Copenhagen University Hospital - North Zealand, Denmark. Patients were enrolled at the Emergency Department and medical wards between January 2019 and April 2021 and followed for 90 days after admission. The exposure was adiponectin measured at baseline.

### 2.3 Participants

In Surviving Pneumonia, all patients hospitalized with a suspected lower respiratory tract infection were screened for eligibility. The eligibility criteria were age ≥ 18 years, new infiltrate on chest x-ray or chest computed tomography scan and at least one symptom or clinical sign consistent with pneumonia, e.g., cough, sputum production, fever (≥38.0°C), hypothermia (<35.0°C), chest pain, breathlessness, and abnormal chest auscultation. The exclusion criterion for the present study was a positive polymerase chain reaction test for severe acute respiratory syndrome coronavirus 2 (SARS-CoV-2). Patients were enrolled within 24 h of admission.

### 2.4 Ethical considerations

The study was approved by the Scientific Ethics Committee at the Capital Region of Denmark (H-18024256), registered on ClinicalTrials.gov (NCT03795662), and conducted in accordance with the Declaration of Helsinki (23). Oral and written informed consent was obtained from all patients before enrolment.

### 2.5 Data collection and variables

Data were collected prospectively with standardized forms and entered into a REDCap database by a few experienced nurses to reduce information bias (24). Information about demography, past medical history, comorbidities, radiology reports, vital signs at admission, blood test results, microbiological test results, and clinical outcomes were collected during a structured interview at study enrolment and from the electronic medical record during the follow-up period. The comorbidity burden was assessed with the Charlson comorbidity index (CCI) (2). Low, medium and high CCI was defined as an index score of 0, 1–2, and ≥3, respectively (2). CAP severity was assessed with the CURB-65 score based on level of confusion, respiratory rate, systolic or diastolic blood pressure and age (25). Clinical stability was defined according to the modified Halm’s criteria when temperature ≤ 37.2°C, heart rate ≤ 100 beats/min, respiratory rate ≤ 24 breaths/min, systolic blood pressure ≥ 90 mm Hg and oxygen saturation ≥ 90% without supplemental oxygen therapy (or habitual oxygen) and quantified in hours since admission (26).

### 2.6 Anthropometric and body composition data

Anthropometric measurements and body composition estimation were performed 24 h after study enrolment. BMI was calculated from self-reported height in meters and weight measured to the nearest 0.1 kg on an electric scale (Seca, Hamburg, Germany). Waist circumference was measured midway between the lowest portion of the rib cage and the uppermost portion of the iliac crest at the umbilical level. Underweight, normal weight, overweight and obesity were defined as BMI < 18.5, 18.5–24.9, 25.0–29.9 and >30 kg/m^2^, respectively. Body fat percentage (%) was estimated with bioimpedance analysis (BioScan touch i8 STD, Maltron International, United Kingdom) using multi-frequency (5, 50, 100, and 200 kHz) hand-to-foot measurements of resistance and reactance with the patient in a supine position.

### 2.7 Adiponectin and routine blood samples

Blood samples were collected in serum tubes and centrifuged at 3,000 x *g* for 15 min at 4°C. The supernatant was stored at −80°C. Serum adiponectin was measured with a commercially available immunoassay (R-PLEX^®^ Human Adiponectin Antibody Set, Meso Scale Diagnostics, Rockville, Maryland, United States) on an electrochemiluminescent platform (Meso Scale Diagnostics, Rockville, Maryland, United States) according to the manufacturer’s instructions. All samples were run in duplicate. The inter-and intra-assay coefficient of variation was <8%. Standard blood tests such as c-reactive protein (CRP) and plasma glucose were performed daily at the attending physician’s discretion.

### 2.8 Outcome measures and confounders

The primary outcome was 90-day mortality. The secondary outcomes were associations of adiponectin with baseline characteristics, in-hospital mortality, the need for non-invasive respiratory support (high flow oxygen therapy, continuous positive airway pressure therapy), intensive care unit (ICU) transferral, time to clinical stability, length of stay (LOS), and 90-day readmission. Age, sex, and adiposity measures were included as confounders in all analyses, with adiponectin as a predictor according to the directed acyclic graphs of the expected causal relationships (Supplementary Figure 1).

### 2.9 Statistical analyses

The continuous variables were skewed and reported as medians [interquartile range (IQR)]. Categorical variables were reported as counts and percentages. As BMI influences adiponectin levels (27), baseline characteristics of patients with and without a BMI measurement were compared. Comparisons of 2 independent samples were performed with Mann–Whitney *U* tests. Kruskal–Walli’s test was used for more than two independent samples, followed by Dunns’s *post hoc* test for multiple comparisons. Differences in categorical variables were estimated with the chi-squared test.

The study participants were divided into four groups based on the sex-stratified quartile distribution of baseline adiponectin because adiponectin levels are higher in females than males (9). Cumulative mortality during the follow-up period across the adiponectin quartiles was assessed with Kaplan–Meier curves and log-rank tests.

Associations between baseline characteristics and adiponectin were modeled with univariate and multivariate linear regression adjusted for age, sex, BMI, select comorbidities (diabetes mellitus, COPD, ischemic heart disease, heart failure, chronic kidney disease, cancer, rheumatic disease), CURB-65 score, and admission CRP level.

The association of continuous outcome variables with adiponectin was estimated with linear regression. Continuous outcome variables, including adiponectin, were skewed and log-transformed to satisfy linear regression model assumptions. The reported exponentiated *β*-coefficients with 95% CI represent the fold change. Logistic regression was used to estimate the association of categorical outcome variables with adiponectin and reported as odds ratios (OR) with 95% CI. The regression models for all outcomes except where adiponectin was the outcome variable were adjusted for age and sex (minimally adjusted models), followed by subsequent adjustments for BMI (fully adjusted models). Adiponectin was evaluated as a continuous variable and quartiles in these models.

Patients who died in-hospital were excluded from analyses where the outcome variable was 90-day readmission, LOS, or time clinical stability. In addition, patients with a do-not-resuscitate order or limitations on life-sustaining treatment were excluded from analyses where ICU admission was the outcome variable.

Complete case analysis was performed in case of missing independent variables in the regression analyses. Sensitivity analyses were performed by entering waist circumference or body fat % in the regression models instead of BMI as more precise measures of adiposity (8). BMI was the primary measure of adiposity because more patients had an available BMI measurement than waist circumference or fat percentage. The statistical comparisons were 2-sided, with *p* < 0.05 as the significance level. The statistical analyses were conducted with R software (R version 4.0.3).

## 3 Results

### 3.1 Patient characteristics

**Figure 1:**
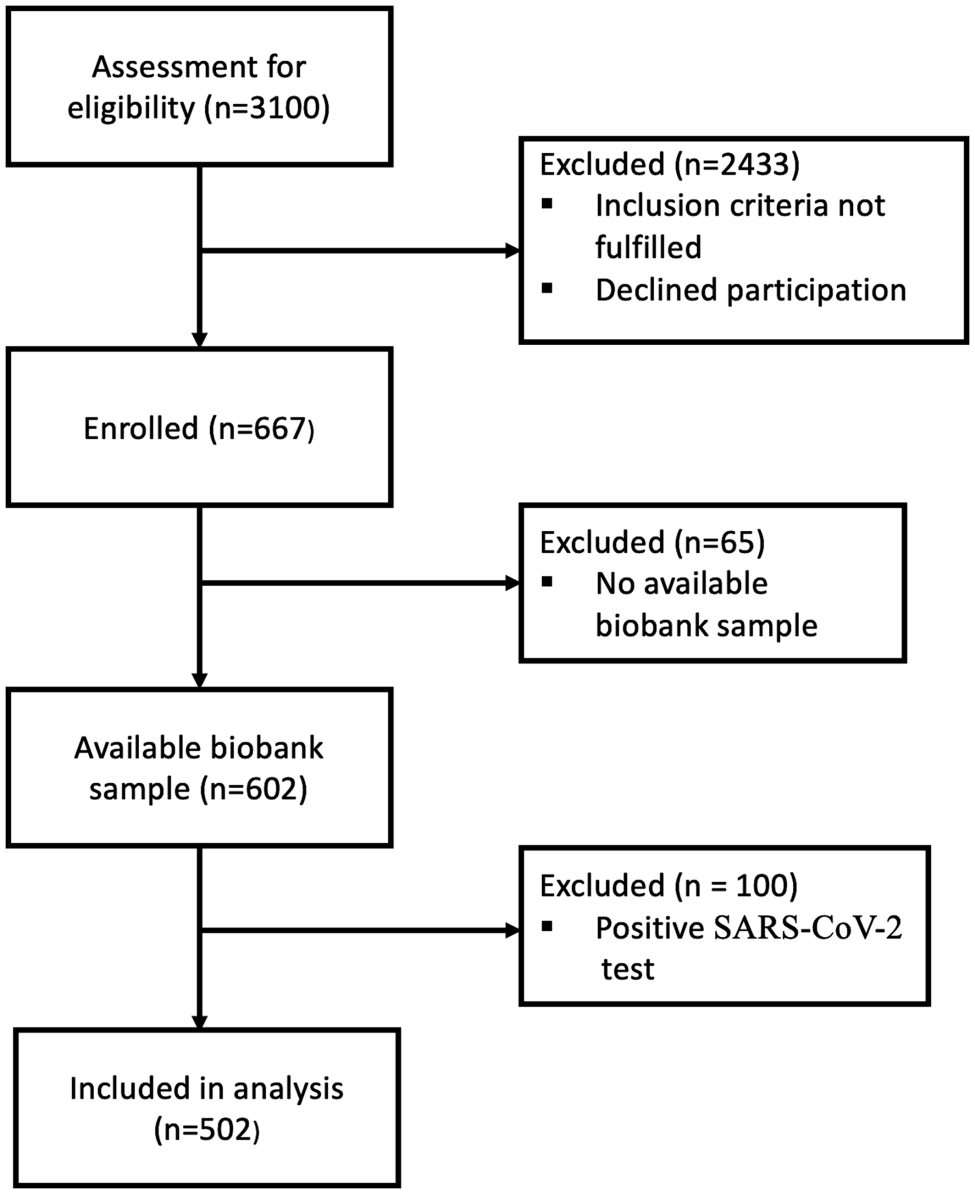
Flow chart of patient enrolment. 2,433 were excluded because they did not fulfill all inclusion criteria. SARS-CoV-2, severe acute respiratory syndrome coronavirus 2.

Among the 3,100 patients screened for eligibility, 502 fulfilled the inclusion criteria and were included (Figure 1). Patient characteristics are shown in Table 1. The median age was 74 years (IQR, 65–81), 50% were female, 6.2% were underweight, 58% were overweight or obese, and 80% had a CCI score ≥ 3. Furthermore, 38, 20, and 19% of the patients had been diagnosed with COPD, cancer, or diabetes, respectively. Patients with no BMI measurement (20%) were older and had higher CCI scores, disease severity, and mortality rate than those with a BMI (Table 2). Adiponectin levels were similar in patients with and without a BMI measurement [median 19 (IQR, 13–27) μg/mL vs. 22 (IQR, 13–30) μg/mL, *p* = 0.2].

**Table 1:** Baseline characteristics and clinical outcomes of 502 patients hospitalized with community-acquired pneumonia.

Variable	Median (IQR) or *n* (%)
Demography	
Age, years	74 (65–81)
Sex, female	249 (50)
Anthropometry	
Body mass index (kg/m^2^)^#^	26 (22–30)
Waist circumference (cm)^#^	100 (90–110)
Body fat percentage^#^	29 (23–38)
Comorbidities	
Diabetes	94 (19)
COPD	189 (38)
Ischemic heart disease	48 (9.6)
Heart failure	78 (16)
Chronic kidney disease	15 (3.0)
Cancer	97 (20)
Rheumatic disease	30 (6.0)
Charlson comorbidity index	5 (3–6)
Disease severity	
CURB-65 score	
Mild (0–1)	269 (54)
Moderate (2)	173 (34)
Severe (3–5)	60 (12)
Baseline blood biomarkers	
Serum adiponectin (μg/ml)	19 (13–28)
C-reactive protein (mg/l)	106 (45–174)
Clinical outcomes	
Primary	
90-day mortality	76 (15)
Secondary	
In-hospital mortality	32 (6.4)
ICU transferral	16 (3.2)
Mechanical ventilation	8 (1.6)
CPAP or high-flow oxygen therapy	54 (11)
Length of stay (days)	5 (4–9)
Time to clinical stability (hours)	100 (50–175)
90-day readmission	168 (33)

^#^Missing data: BMI 20%, body fat percentage 38%, waist circumference 41%. COPD, chronic obstructive pulmonary disease; CURB-65 score is based on 5 variables: confusion, urea >7 mmol/L, respiratory rate ≥30 breaths/min, systolic blood pressure <90 mmHg or diastolic blood pressure ≤60 mmHg and age ≥65 years; CPAP, continuous positive airway pressure; hrs, hours; ICU, intensive care unit; IQR, interquartile range.

**Table 2:** Patient characteristics and clinical outcomes according to the availability of a BMI measurement.

Characteristic	*N*	No BMI *N* = 102	BMI available *N* = 400	*p*-value^1^
Demography				
Age, years	502	78 (70, 85)	73 (64, 80)	0.003
Sex, female	502	46 (45%)	203 (51%)	0.3
Comorbidities				
Charlson comorbidity index	491	5 (4, 7)	4 (3, 6)	0.006
Disease severity				
CURB-65 score	502			<0.001
Mild (0–1)		39 (38%)	230 (57%)	
Moderate (2)		42 (41%)	131 (33%)	
Severe (3–5)		21 (21%)	39 (9.8%)	
Clinical outcomes				
Primary				
Non-survivor	502	23 (23%)	53 (13%)	0.019
Secondary				
In-hospital mortality	492	11 (11%)	21 (5.3%)	0.028
ICU-admission	502	6 (6.2%)	20 (5.1%)	0.6
CPAP or high-flow O_2_	502	9 (8.8%)	45 (11%)	0.5
Length of stay (days)	502	5 (4, 8)	5 (4, 9)	0.3
Time to clinical stability (hours)	495	92 (41, 162)	102 (53, 179)	0.2
90-day readmission	502	27 (26%)	141 (35%)	0.093

^1^Mann–Whitney *U* tests test; Pearson’s Chi-squared test. COPD, chronic obstructive pulmonary disease; CURB-65 score is based on 5 variables: confusion, urea >7 mmol/L, respiratory rate ≥30 breaths/min, systolic blood pressure <90 mmHg or diastolic blood pressure ≤60 mmHg and age ≥65 years; CPAP, continuous positive airway pressure; hrs, hours; ICU, intensive care unit; IQR, interquartile range.

### 3.2 Association between adiponectin and baseline characteristics

**Figure 2:**
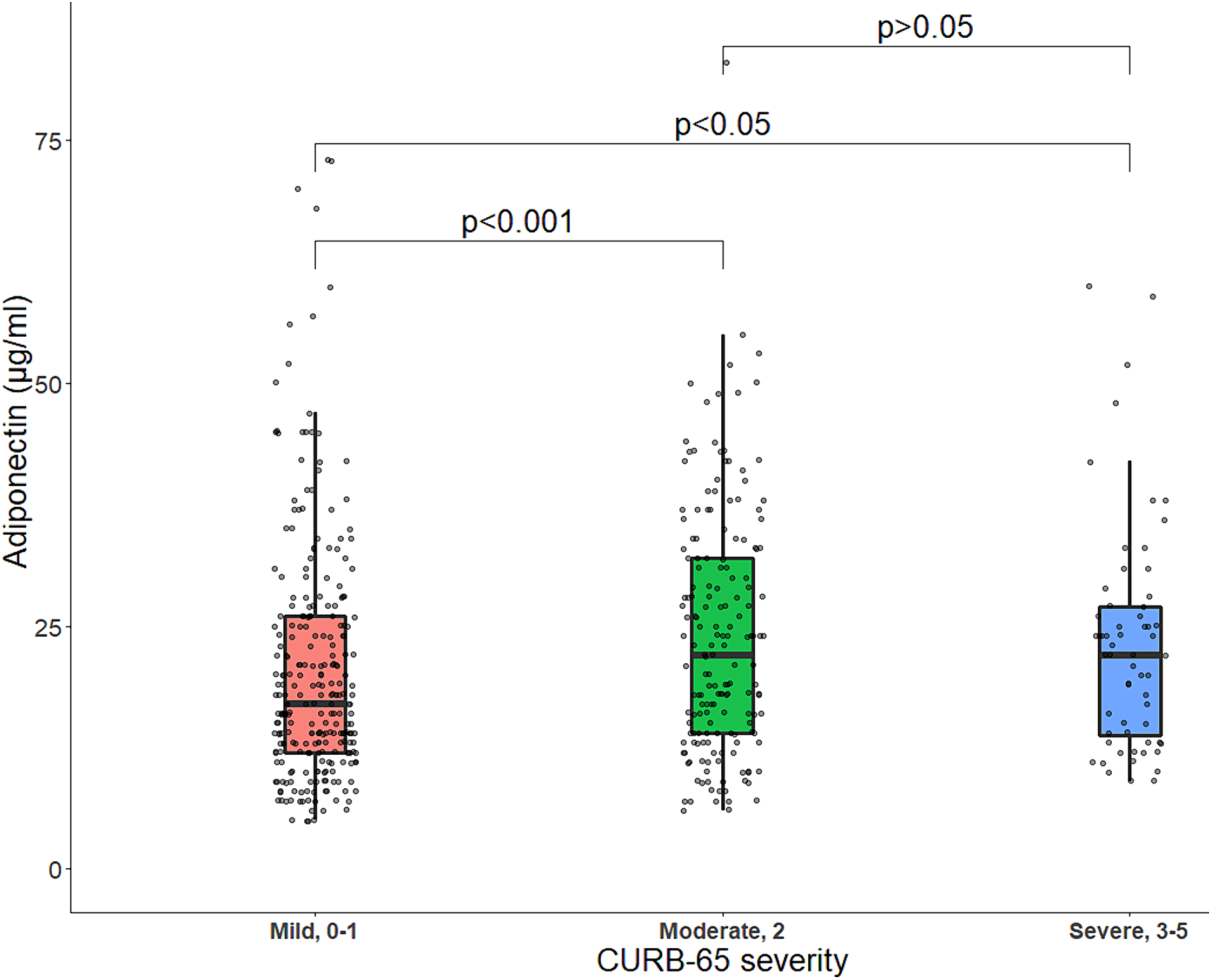
Boxplot of baseline serum adiponectin levels (μg/mL) stratified by CURB-65 score. The line within the box represents the median serum adiponectin levels, and each box represents the upper and lower quartiles. The jittered points around each boxplot represent the distribution of adiponectin. Statistical comparisons were made with Mann–Whitney *U* tests for two-group comparisons or Kruskal–Wallis tests in case of more than two independent samples, followed by Dunns’s *post hoc* test for multiple comparisons. p, *p*-value.

Patients presenting with moderate or severe disease, according to the CURB-65 score, had higher adiponectin levels than patients with mild disease (Figure 2). In multivariate linear regression analyses, increasing age, female sex, having COPD, and rheumatic disease were associated with higher adiponectin levels, whereas increasing BMI, having diabetes mellitus, and higher admission CRP were associated with lower adiponectin levels (Table 3).

**Table 3:** Linear regression analyses of the associations between baseline characteristics and adiponectin levels.

	Univariate models			Multivariate model		
Predictors	*β*	95% CI	*p*-value	*β*	95% CI	*p*-value
Waist circumference (cm)^a^	0.99	0.98–0.99	<0.001			
Body fat %^b^	0.99	0.99–1.00	0.03			
BMI (kg/m^2^)^c^	0.97	0.96–0.98	<0.001	0.98	0.97–0.99	<0.001
Age years	1.01	1.01–1.02	<0.001	1.01	1.00–1.01	0.001
Sex (female)	1.24	1.13–1.37	<0.001	1.28	1.17–1.40	<0.001
Diabetes	0.74	0.66–0.84	<0.001	0.80	0.71–0.91	0.001
COPD	1.36	1.24–1.50	<0.001	1.22	1.10–1.34	<0.001
Ischemic heart disease	1.02	0.86–1.20	0.837	0.90	0.76–1.07	0.220
Heart failure	1.14	1.00–1.30	0.053	1.04	0.91–1.19	0.544
Chronic kidney disease	1.12	0.84–1.48	0.443	1.24	0.95–1.63	0.114
Cancer	1.13	1.00–1.28	0.044	1.02	0.90–1.15	0.757
Rheumatic disease	1.32	1.08–1.62	0.007	1.24	1.03–1.50	0.023
CRP (50 mg/L increase)	0.94	0.91–0.96	<0.001	0.94	0.92–0.96	<0.001
CURB-65 score	1.11	1.05–1.16	<0.001	1.03	0.97–1.10	0.322

Data are from linear regression analyses with log-transformed adiponectin as the dependent variable. Data are reported as fold change in regression coefficient (β) after back transformation. BMI, body mass index; COPD, chronic obstructive pulmonary disease; CRP, c-reactive protein; CURB-65 score is based on 5 variables: confusion, urea >7 mmol/L, respiratory rate ≥30 breaths/min, systolic blood pressure <90 mmHg or diastolic blood pressure ≤60 mmHg and age ≥65 years.

^a^Based on 296 observations.

^b^Based on 311 observations.

^c^Based on 400 observations.

### 3.3 90-day mortality (primary endpoint)

**Figure 3:**
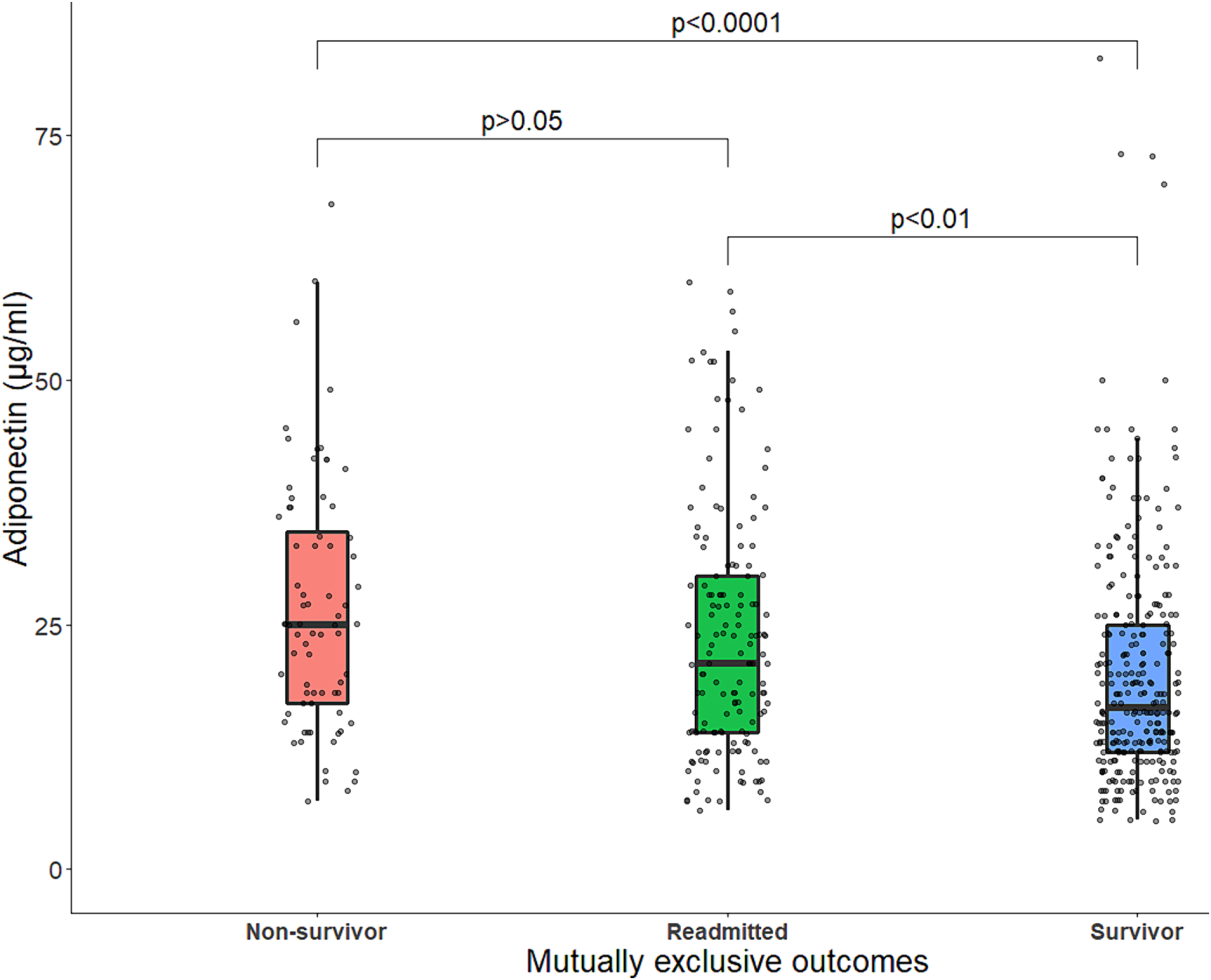
Boxplot of baseline serum adiponectin levels (μg/mL) stratified by outcome status. The line within the box represents the median serum adiponectin levels, and each box represents the upper and lower quartiles. The jittered points around each boxplot represent the distribution of adiponectin. Statistical comparisons were made with Mann–Whitney *U* tests for two-group comparisons or Kruskal–Wallis tests in case of more than two independent samples, followed by Dunns’s post-hoc test for multiple comparisons. p, *p*-value.

**Figure 4:**
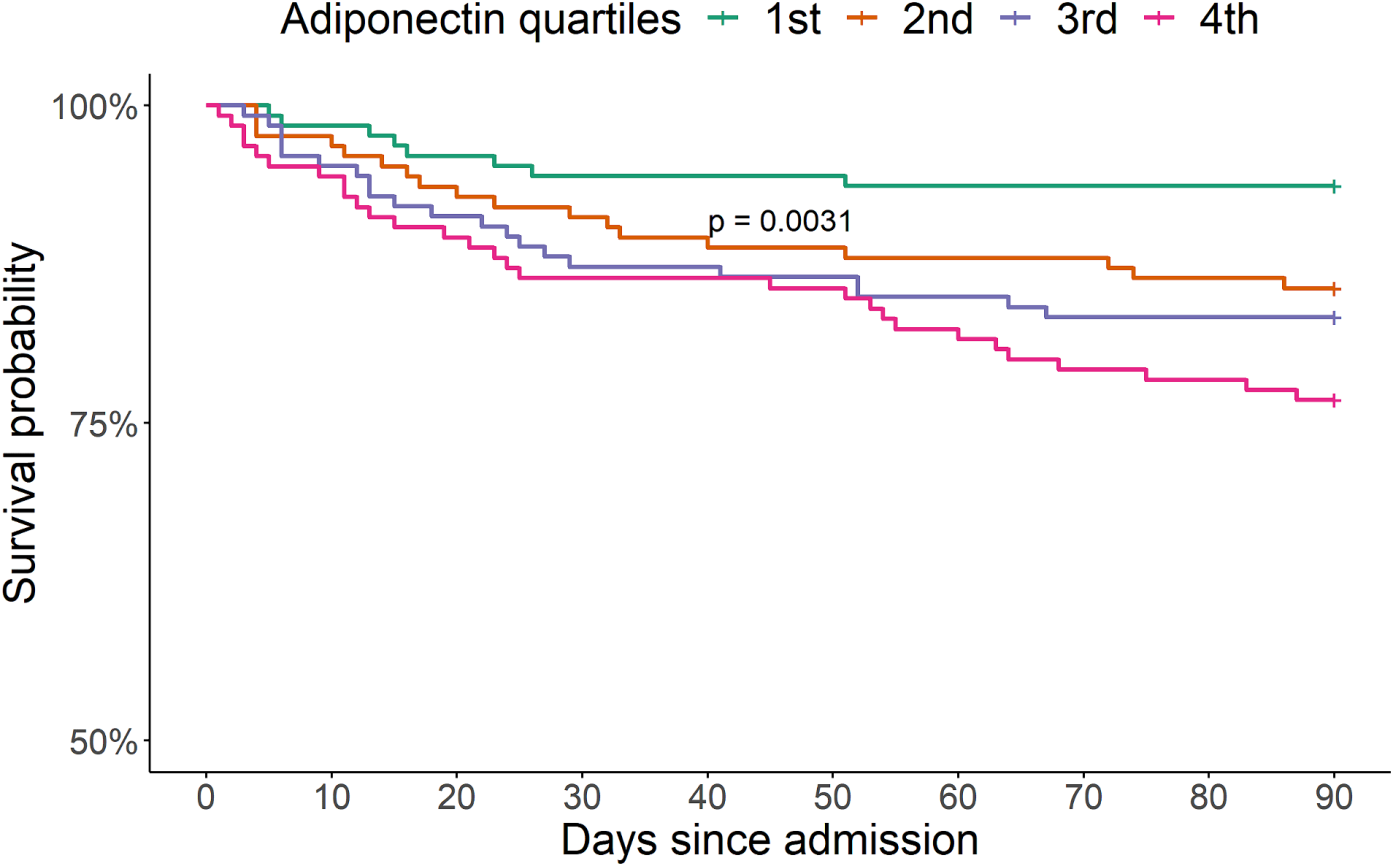
Kaplan–Meier survival probability curves 90 days after admission according to sex-adjusted adiponectin quartiles for 502 patients with community-acquired pneumonia. The survival curves for the second, third and fourth quartiles were significantly different from the first quartile according to a log-rank test (*p* = 0.0031).

Adiponectin levels were higher in non-survivors compared to survivors (Figure 3). The survival curves showed a decreasing survival probability with increasing adiponectin quartiles (*p* < 0.003) (Figure 4). The 90-day survival probability was 94% (95% CI: 91–98%) for patients with adiponectin levels in the 1st quartile, 87% (95% CI: 82–94%) for those in the 2nd quartile, 80% (95% CI: 73–88%) for the 3rd quartile, and 76% (95% CI: 69–84%) for the 4th quartile (Figure 3). Pairwise log-rank tests showed that the survival probability for patients with adiponectin levels in the 4th quartile was significantly lower than for those in the 1st (*p* < 0.001) and 2nd quartiles (*p* = 0.048), and for patients in the 3rd quartile compared to the 1st (*p* = 0.001).

In minimally adjusted models, the odds ratio (OR) for 90-day mortality increased by 2% for each 1 μg/mL increase in adiponectin and was 199 and 234% higher for adiponectin levels in the 3rd and 4th quartiles, respectively, than in the 1st quartile (Table 4). When BMI was added to the analysis (the fully adjusted model), the association between adiponectin and 90-day mortality disappeared (Table 4). Likewise, the association disappeared when adjusted for waist circumference (*n* = 296) or body fat % (*n* = 311) (Table 5).

**Table 4:** Logistic regression analyses of the association between adiponectin levels at admission and 90-day mortality, in-hospital mortality, and readmission.

Outcomes	90-day mortality						In-hospital mortality						90-day readmission					
	Minimally adjusted^1^			Fully adjusted^2^			Minimally adjusted^1^			Fully adjusted^2^			Minimally adjusted^1^			Fully adjusted^2^		
Predictors	OR	95% CI	*p*-value	OR	95% CI	*p*-value	OR	95% CI	*p*-value	OR	95% CI	*p*-value	OR	95% CI	*p*-value	OR	95% CI	*p*-value
Adiponectin μg/ml																		
Continuous	1.02	1.00–1.04	0.048	1.01	0.98–1.03	0.52	1.02	0.99–1.04	0.21	0.99	0.95–1.03	0.60	1.02	1.01–1.04	0.007	1.02	1.00–1.04	0.024
1st quartile	Ref.			Ref.			Ref.			Ref.			Ref.					
2nd quartile	1.96	0.79–5.19	0.15	1.87	0.68–5.54	0.23	3.65	0.85–25.05	0.11	3.66	0.84–25.40	0.12	1.25	0.71–2.20	0.44	1.15	0.63–2.11	0.64
3rd quartile	2.99	1.29–7.59	0.014	1.57	0.59–4.52	0.38	5.82	1.47–38.37	0.025	2.38	0.52–16.84	0.30	1.87	1.07–3.27	0.028	1.47	0.79–2.75	0.23
4th quartile	3.34	1.46–8.38	0.006	2.01	0.77–5.70	0.17	4.65	1.18–30.96	0.06	1.56	0.31–11.56	0.61	2.33	1.34–4.09	0.003	2.24	1.20–4.23	0.012

Patients who died in the hospital were excluded from analyses where readmission was the outcome. BMI, body mass index; OR, odds ratio; CI, confidence interval; Ref., reference.

^1^Models were adjusted for age and sex.

^2^Models were adjusted for age, sex, and BMI.

^3^Adiponectin quartile ranges for females: <15 μg/mL, 15–21 μg/mL, 21.1–31 μg/mL, and >31 μg/mL for the first, second, third, and fourth quartile, respectively. The corresponding ranges for males: <12 μg/mL, 12–17 μg/mL, 17.1–26 μg/mL, and > 26 μg/mL for the first, second, third, and fourth quartile, respectively.

**Table 5:** Logistic regression models of the association between adiponectin levels and 90-day mortality, adjusted for waist circumference or body fat %.

Outcome	90-day mortality					
	Fully adjusted^1^ (waist circumference, cm)			Fully adjusted^1^ (body fat %)		
Predictors	OR	95% CI	*p*-value	OR	95% CI	*p*-value
Adiponectin μg/ml						
Continuous	1.01	0.98–1.04	0.67	1.02	0.99–1.04	0.24
^2^1st quartile	Ref.			Ref.		
2nd quartile	1.23	0.36–4.29	0.74	1.56	0.46–5.75	0.48
3rd quartile	1.31	0.41–4.42	0.65	1.44	0.44–5.16	0.55
4th quartile	1.31	0.42–4.35	0.65	2.33	0.77–7.99	0.15

^1^Models were also adjusted for age and sex.

^2^Adiponectin quartile ranges for females: <15 μg/mL, 15–21 μg/mL, 21.1–31 μg/mL, and >31 μg/mL for the first, second, third, and fourth quartile, respectively. The corresponding ranges for males: <12 μg/mL, 12–17 μg/mL, 17.1–26 μg/mL, and > 26 μg/mL for the first, second, third, and fourth quartile, respectively. Ref., reference, OR, odds ratio; CI, confidence interval.

### 3.4 In-hospital mortality

**Figure 5:**
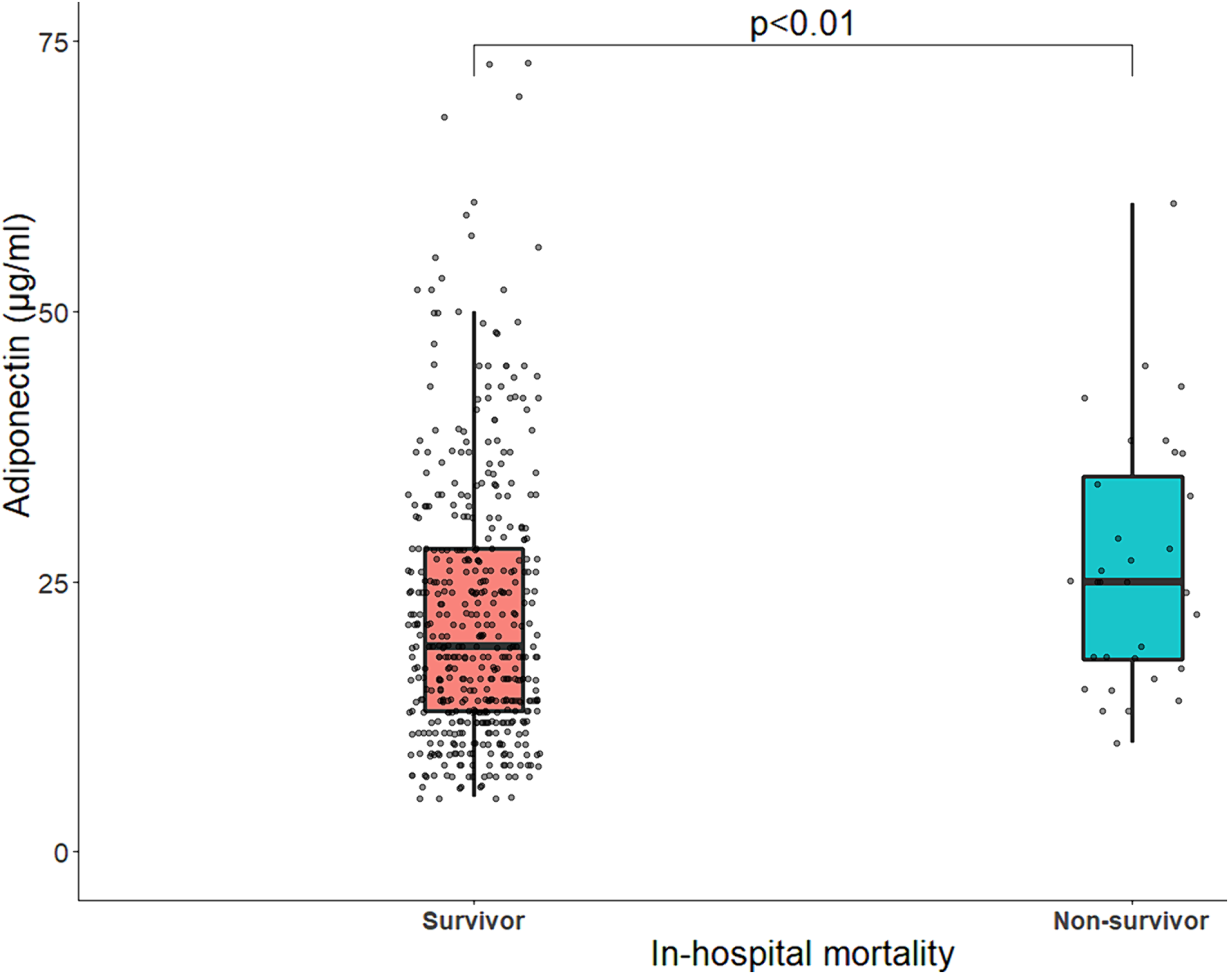
Boxplot of baseline serum adiponectin levels (μg/mL) stratified by in-hospital mortality. The line within the box represents the median serum adiponectin levels, and each box represents the upper and lower quartiles. The jittered points around each boxplot represent the distribution of adiponectin. Statistical comparisons were made with Mann–Whitney U tests for two-group comparisons. p, *p*-value.

Overall, 6.4% (*n* = 32) of the patients died in hospital. Their adiponectin levels were higher than those of patients who survived until discharge (Figure 5). The OR for in-hospital mortality was 482 and 365% higher for adiponectin levels in the 3rd and 4th quartiles, respectively, than in the 1st quartile in minimally adjusted models (Table 4). In the fully adjusted model, the association between adiponectin and in-hospital mortality disappeared (Table 4).

### 3.5 ICU transferral, non-invasive respiratory support, length of stay and time to clinical stability

There were no associations between adiponectin levels and risk of ICU transferral, non-invasive respiratory support (Table 6), LOS or time to clinical stability in the minimally or fully adjusted models (Table 7).

**Table 6:** Logistic regression models of the association between adiponectin levels, ICU admission and non-invasive respiratory support.

Outcome	ICU admission						Non-invasive respiratory support					
	Minimally adjusted^1^			Fully adjusted^2^			Minimally adjusted^1^			Fully adjusted^2^		
Predictors	OR	95% CI	*p*-value	OR	95% CI	*p*-value	OR	95% CI	*p*-value	OR	95% CI	*p*-value
Adiponectin μg/ml												
Continuous	1.00	0.96–1.04	0.80	0.99	0.93–1.03	0.59	1.01	0.98–1.03	0.52	1.00	0.97–1.03	0.97
^3^1st quartile	Ref.						Ref.					
2nd quartile	1.09	0.26–4.35	0.90	1.00	0.24–4.01	1.00	1.13	0.48–2.64	0.77	1.02	0.42–2.48	0.96
3rd quartile	3.00	0.98–10.37	0.063	1.87	0.54–7.10	0.34	1.44	0.64–3.30	0.38	0.81	0.31–2.07	0.66
4th quartile	0.94	0.18–4.22	0.94	0.59	0.08–3.15	0.56	1.31	0.56–3.07	0.53	1.06	0.42–2.71	0.90

Patients with a do-not-resuscitate order or limitations on life-sustaining treatment were excluded from analyses where ICU admission was the outcome variable. OR, odds ratio; CI, confidence interval; Ref., reference.

^1^Models were adjusted for age and sex.

^2^Models were adjusted for age, sex, and BMI.

^3^Adiponectin quartile ranges for females: <15 μg/mL, 15–21 μg/mL, 21.1–31 μg/mL, and >31 μg/mL for the first, second, third, and fourth quartile, respectively. The corresponding ranges for males: <12 μg/mL, 12–17 μg/mL, 17.1–26 μg/mL, and >26 μg/mL for the first, second, third, and fourth quartile, respectively.

**Table 7:** Linear regression models of the association between adiponectin levels and log-transformed length of stay and time to clinical stability.

Outcomes	Length of stay						Time to clinical stability					
	Minimally adjusted^1^			Fully adjusted^2^			Minimally adjusted^1^			Fully adjusted^2^		
Predictors	β	95% CI	*p*-value	β	95% CI	*p*-value	β	95% CI	*p*-value	β	95% CI	*p*-value
Adiponectin μg/ml												
Continuous	1.00	0.99–1.00	0.20	0.99	0.99–1.00	0.11	0.99	0.98–1.00	0.055	0.99	0.98–1.00	0.152
^3^1st quartile	Ref.			Ref.			Ref.			Ref.		
2nd quartile	0.97	0.78–1.20	0.75	0.93	0.73–1.19	0.58	0.89	0.66–1.21	0.47	0.88	0.64–1.22	0.44
3rd quartile	0.90	0.72–1.13	0.36	0.84	0.65–1.08	0.17	0.88	0.64–1.19	0.40	0.80	0.57–1.12	0.20
4th quartile	0.93	0.75–1.16	0.53	0.91	0.71–1.18	0.49	0.87	0.64–1.19	0.39	0.85	0.60–1.20	0.35

Data are from linear regression analyses with the log-transformed length of stay and time to clinical stability as the dependent variables. Patients who died during admission were excluded from these analyses. Data are reported as fold change in regression coefficient (β) after back transformation. Ref., reference; CI, confidence interval.

^1^Models were adjusted for age and sex.

^2^Models were adjusted for age, sex, and BMI.

^3^Adiponectin quartile ranges for females: <15 μg/mL, 15–21 μg/mL, 21.1–31 μg/mL, and > 31 μg/mL for the first, second, third, and fourth quartile, respectively. The corresponding ranges for males: <12 μg/mL, 12–17 μg/mL, 17.1–26 μg/mL, and >26 μg/mL for the first, second, third, and fourth quartile, respectively.

### 3.6 90-day readmission

33.5% (*n* = 168) of the patients were readmitted within 90 days. Their adiponectin levels were higher than those of patients who were not readmitted (Figure 3). Exacerbation of chronic diseases and infections were the most common causes of readmission (Table 8). The OR for 90-day readmission increased by 2% for each μg/mL increase in adiponectin and was 87 and 133% higher for adiponectin levels in the 3rd and 4th quartiles, respectively, than the 1st quartile in minimally adjusted models. This association remained in the fully adjusted model when adiponectin was entered as a continuous variable and for adiponectin levels in the 4th quartile (Table 4). The results were similar when BMI was substituted for waist circumference or body fat % (Table 9).

**Table 8:** Overall causes of readmission.

Disease category	*N* (%)
^1^Miscellaneous acute conditions	67 (40)
^2^Exacerbation of chronic disease	52 (40)
^3^Respiratory infection	35 (21)
^4^Other infections	14 (8)

^1^Includes acute medical and surgical conditions (and not exacerbation of chronic diseases).

^2^For example, acute exacerbation or complication due to COPD, asthma, cancer, cardiovascular disease, neurological disease.

^3^For example, pneumonia, viral respiratory infection.

^4^For example, endocarditis, urinary tract infections, skin and soft-tissue infections.

**Table 9:** Logistic regression models of the association between adiponectin levels and 90-day readmission adjusted for waist circumference or body fat %.

Outcome	90-day readmission					
	Fully adjusted^1^ (Waist circumference, cm)			Fully adjusted^2^ (Body fat %)		
Predictors	OR	95% CI	*p*-value	OR	95% CI	*p*-value
Adiponectin μg/ml						
Continuous	1.03	1.00–1.05	0.023	1.02	1.00–1.04	0.031
^3^1st quartiles	Ref.			Ref.		
2nd quartile	1.22	0.59–2.50	0.589	0.97	0.50–1.89	0.936
3rd quartile	1.71	0.83–3.57	0.147	1.31	0.67–2.57	0.427
4th quartile	2.26	1.06–4.87	0.036	2.01	1.01–4.07	0.048

Patients who died in hospital were excluded from these analyses. Ref., reference; OR, odds ratio; CI, confidence interval.

^1^Models were adjusted for age, sex, and waist circumference (cm).

^2^Models were adjusted for age, sex, and body fat %.

^3^Adiponectin quartile ranges for females: <15 μg/mL, 15–21 μg/mL, 21.1–31 μg/mL, and >31 μg/mL for the first, second, third, and fourth quartile, respectively. The corresponding ranges for males: <12 μg/mL, 12–17 μg/mL, 17.1–26 μg/mL, and >26 μg/mL for the first, second, third, and fourth quartile, respectively.

## 4 Discussion

Our study is the first to investigate whether adiponectin is associated with adverse clinical outcomes after hospitalization with CAP. We found a positive association between high adiponectin levels and 90-day mortality independent of age and sex in a dose–response-like relationship for adiponectin quartiles. The association disappeared in analyses adjusted for BMI and specific adiposity measurements such as waist circumference or fat percentage. Finally, high adiponectin levels were associated with an increased risk of 90-day readmission independent of age, sex, and adiposity.

In line with previous studies, we found that increasing age, female sex, presence of COPD and rheumatic disease, absence of diabetes mellitus, low CRP, and low BMI were independent predictors of higher adiponectin levels (9, 21, 28, 29), whereas the CURB-65 score was not. This finding suggests that disease severity does not independently affect adiponectin levels, supporting other studies in critically ill patients (21, 30, 31).

Our findings suggest that low body fat explained the association between high adiponectin levels and increased mortality risk.

Consistent with our findings, two studies of critically ill patients with sepsis demonstrated an association between high adiponectin levels and increased risk of 28-day and 30-day mortality in unadjusted analyses that disappeared in models adjusted for BMI (32, 33). In contrast, two other studies of critically ill patients with respiratory failure and sepsis found an association between high adiponectin levels and increased risk of 28-day and 60-day mortality independent of BMI (20, 21). In a study of patients with acute respiratory distress syndrome (ARDS), high adiponectin levels were associated with an increased risk of 60-day mortality independent of BMI only in a subgroup of patients with an extra-pulmonary cause of ARDS. However, there was no association in the entire cohort (34).

Compared to our study population, the patients in the cited studies were critically ill, younger, predominantly male, more ethnically diverse, more obese, and more frequently diagnosed with diabetes mellitus (20, 21, 32–34). Furthermore, none of the cited studies had body composition or waist circumference data and could, therefore, not assess adiponectin’s association with mortality accounting for the amount of adipose tissue or abdominal obesity. Another complicating factor is that the etiology of critical illness was heterogeneous across the cited studies, where only a minority were diagnosed with pneumonia (20, 21, 32–34). These differences in patient characteristics might explain why adiponectin was a predictor of mortality independent of BMI in some studies. As an outlier, a study showed that sepsis non-survivors had lower adiponectin levels than survivors (19). However, the authors did not include age, sex, or BMI in the adjusted analyses, a significant limitation of this study (19).

A limitation of using 90-day mortality as an outcome measure after CAP is that the proportion of deaths directly attributed to pneumonia decreases while the proportion of deaths secondary to underlying diseases increases over time (35). We found an association between adiponectin and in-hospital mortality in models adjusted for age and sex, suggesting that high adiponectin levels can predict mortality more likely directly attributed to CAP. However, the association was weak, with wide confidence intervals, indicating a small sample size.

Among the patients who survived until discharge, we found no association between adiponectin levels and adverse clinical outcomes during hospitalization. Therefore, our results suggest that adiponectin is not associated with a more severe or protracted course of CAP. Our findings are supported by a small study of critically ill patients with SARS-CoV2 and non-SARS-CoV2 pneumonia that found no association between adiponectin levels and duration of mechanical ventilation (31).

Our study is the first to show that high adiponectin levels are associated with an increased risk of readmission after an acute hospitalization for CAP. Furthermore, the association was independent of adiposity. Readmission after CAP is often due to worsening pre-existing diseases, as shown by our data and others (36). In line with our results, a study of patients with heart failure showed an increased risk of mortality and readmission independent of BMI with increasing adiponectin levels (37). However, a limitation of this study was the use of mortality and readmission as a composite outcome (37). Adiponectin resistance may explain why high adiponectin levels are associated with worse outcomes in heart failure (18, 38). These considerations could be relevant to our findings. Future studies should investigate whether adiponectin can predict specific causes of readmissions after CAP. Our findings suggest that high adiponectin levels in hospitalized patients with CAP may reflect the negative influence of aging and comorbidities on body composition, specifically low body fat mass. Therefore, we speculate that high adiponectin may be a biomarker of adverse body composition associated with mortality and readmission in patients with CAP. However, our findings need to be replicated and validated in different settings.

### 4.1 Strengths and limitations

The prospective design, standardized data collection and detailed characterization of body composition are strengths of our study. Furthermore, we consider our cohort representative of patients hospitalized with CAP in a high-income, European country since we only had one exclusion criterion.

Nevertheless, our single-center study design limits the generalisability of the results. In addition, we cannot rule out consent bias since the study participants had to give active consent, thus excluding incapacitated patients. Another study limitation was the lack of a BMI measurement in 20% of patients. The patients without a BMI measurement had more severe CAP and thus could not fully participate in the study. Even though patients without a BMI had a higher mortality rate than those with a BMI, we do not expect this to have affected the overall associations because adiponectin levels were similar in both groups but increased the chances of type II error.

## 5 Conclusion

In conclusion, adiponectin was a predictor of mortality and readmission. The association with mortality depended on low body fat, whereas the association with readmission risk was independent of adiposity.

## Data availability statement

The raw data supporting the conclusions of this article will be made available by the authors, upon reasonable request.

## Ethics statement

The studies involving humans were approved by Scientific Ethics Committee at the Capital Region of Denmark. The studies were conducted in accordance with the local legislation and institutional requirements. The participants provided their written informed consent to participate in this study.

## Author contributions

AD: Conceptualization, Data curation, Formal analysis, Funding acquisition, Methodology, Visualization, Writing – original draft, Writing – review & editing. CR: Data curation, Writing – review & editing. MH: Data curation, Writing – review & editing. AS: Data curation, Writing – review & editing. AJ: Writing – review & editing. PK: Writing – review & editing. RK-M: Conceptualization, Methodology, Supervision, Writing – review & editing. DF-J: Conceptualization, Methodology, Supervision, Writing – review & editing. BL: Conceptualization, Funding acquisition, Methodology, Project administration, Resources, Supervision, Writing – review & editing.
