# Corrigendum: Adiponectin as a predictor of mortality and readmission in patients with community-acquired pneumonia: a prospective cohort study

**DOI:** 10.3389/fmed.2024.1420295

**Published:** 2024-05-01

**Authors:** Arnold Matovu Dungu, Camilla Koch Ryrsø, Maria Hein Hegelund, Adin Sejdic, Andreas Vestergaard Jensen, Peter Lommer Kristensen, Rikke Krogh-Madsen, Daniel Faurholt-Jepsen, Birgitte Lindegaard

**Affiliations:** ^1^Department of Pulmonary and Infectious Diseases, Copenhagen University Hospital - North Zealand, Hilleroed, Denmark; ^2^Department of Endocrinology and Nephrology, Copenhagen University Hospital - North Zealand, Hilleroed, Denmark; ^3^Department of Clinical Medicine, University of Copenhagen, Copenhagen, Denmark; ^4^Department of Infectious Diseases, Copenhagen University Hospital - Hvidovre, Copenhagen, Denmark; ^5^Department of Infectious Diseases, Copenhagen University Hospital – Rigshospitalet, Copenhagen, Denmark

**Keywords:** community-acquired pneumonia, adiponectin, body mass index, mortality, readmission

In the published article, there was an error with affiliation 2. Instead of “Department of Endocrinology and Nephrology, Copenhagen University Hospital - North Zealand, Copenhagen, Denmark”, it should be removed. Inclusion of this affiliation was an error.

In the published article, there was an error regarding the affiliations for Rikke Krogh-Madsen. As well as having affiliations 4, they should also have “*Department of Clinical Medicine, University of Copenhagen, Copenhagen, Denmark*”, affiliation 3.

In the published article, there was an error regarding the affiliations for Daniel Faurholt-Jepsen. As well as having affiliations 5, they should also have “*Department of Clinical Medicine, University of Copenhagen, Copenhagen, Denmark*”, affiliation 3.

The authors apologize for this error and state that this does not change the scientific conclusions of the article in any way. The original article has been updated.

