# Supplementary material for: Adiponectin as a predictor of mortality and readmission in patients with community-acquired pneumonia: a prospective cohort study
Source: Front Med (Lausanne). 2024 Apr 2;11:1329417. doi: 10.3389/fmed.2024.1329417 (PMC11022597; doi:10.3389/fmed.2024.1329417)
Supplement: Supplementary file 1 [file Image_1.pdf]

Supplementary Material

1 Supplementary figures

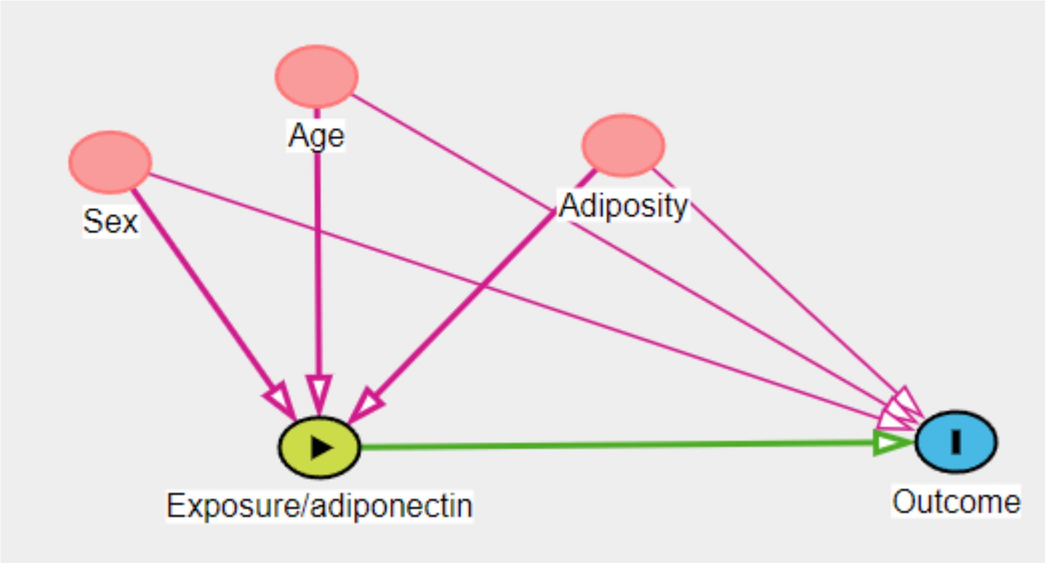

Figure 1: Directed acyclic graph of expected causal relationship between adiponectin and clinical outcomes (90-day mortality, in-hospital mortality, the need for non-invasive respiratory support, intensive care unit transferral, time to clinical stability, length of stay, and 90-day readmission.
